# The Effectiveness of Telerehabilitation on Balance and Functional Mobility in Patients with Stroke: A Systematic Review and Meta-Analysis

**DOI:** 10.5195/ijt.2022.6532

**Published:** 2022-12-13

**Authors:** Mohamed Salaheldien Alayat, Nahla Ahmad Almatrafi, Abdulqader Abdulrazaq Almutairi, Amir Abdel Raouf El Fiky, Ahmed Mohamed Elsodany

**Affiliations:** 1 Department of Physiotherapy, Applied Medical Science College, Umm Al-Qura University, Makkah, Saudi Arabia; 2 Department of Basic Science, Faculty of Physical Therapy, Cairo University, Egypt; 3 Department of Physical Therapy for Neurological Disorders and its Surgery, Faculty of Physical Therapy, Cairo University, Giza, Egypt

**Keywords:** Balance, Functional mobility, Stroke, Telerehabilitation

## Abstract

**Objective:**

The aim of this systematic review and meta-analysis was to investigate the effectiveness of telerehabilitation on improving balance and functional mobility in stroke survivors.

**Methods:**

Comprehensive searching was conducted from inception to May 2022. The inclusion criteria were studies evaluating the effectiveness of telerehabilitation in stroke survivors. Data regarding participants, intervention, outcome measures, and main results were extracted. PEDro scale and the Grading of Recommendations Assessment Development and Evaluation (GRADE) were used to assess the methodological quality and quality of evidence, respectively.

**Data Analysis:**

A total of fourteen articles) 594 patients) were included. A meta-analysis using a random-effect model was performed on thirteen studies )530 patients). Standardized mean difference (SMD) with 95% confidence interval (CI) was calculated for balance and functional mobility. Results: PEDro scale revealed ten good-quality studies, three fair-quality studies, and one poor-quality study. According to the available evidence, telerehabilitation has a small effect size in improving both balance (SMD 0.33 [95% CI 0.03 to 0.63]; P =0.03; low quality of evidence) and functional mobility (SMD 0.27 [95% CI 0.02 to 0.52]; P =0.03; low quality of evidence).

**Conclusion:**

Telerehabilitation may improve balance and functional mobility in stroke survivors. However, it is evident that more high-quality research is required due to the existence of low to very low-quality evidence with limited confidence in the effect estimate.

**Registration:**

PROSPERO registration number (CRD42022306410).

Globally, stroke is one of the major causes of mortality and long-term disabilities (Bevan et al., 2012). One of the most frequent stroke deficits is balance impairment, which makes it difficult for patients to walk safely and increases the risk of falls which can lead to serious injuries and complications (Bambirra et al., 2015), so it is crucial to enhance stroke survivors' ability to regain their balance (Junata et al., 2021). Moreover, a patient's ability to be balanced is necessary to perform tasks like standing, walking, and climbing stairs, hence it plays a significant role in determining one's quality of life (Cho & Kim, 2020).

After hospital discharge, stroke survivors commonly experience secondary physical and functional problems. In addition, they tend to depend on home rehabilitation more than outpatient clinics or institutional rehabilitation, unless a significant health issue develops. This is because of time restraints, resource limitations, geographical remoteness, and the disease itself (Geyh et al., 2007; Wu et al., 2020). Thus, figuring out the best and most efficient ways to provide rehabilitation services should be a matter of concern.

In the past few years, the advancement of computer science technology and telemedical equipment has resulted in a growth in telemedicine and telerehabilitation (Appleby et al., 2019). Telerehabilitation is the delivery of rehabilitation services to patients remotely by using communication technology (Jiang et al., 2018). Various technologies can be used to communicate between the patient and the rehabilitation specialist such as smartphone apps, web-based videoconferencing, etc. (Rogante et al., 2010).

Telerehabilitation services can be utilized to supplement and improve the quality of already available rehabilitation services. Stroke survivors have raised concerns about the absence of long-term assistance and continued unfulfilled rehabilitation needs after discharge from the hospital (Ullberg et al., 2016).

Telerehabilitation has the potential to reduce rehabilitation costs for both therapists and patients, as well as provide an opportunity to obtain rehabilitation services for patients who live in rural and distant areas and those with severely restricted mobility (Peretti et al., 2017). In addition, telerehabilitation can provide therapists with an alternative, innovative, and valuable method of providing rehabilitation to help stroke survivors recover, as well as to adjust and follow their progress remotely (Lloréns et al., 2015).

Telerehabilitation is a method of providing rehabilitation programs, so the mechanisms that lead to recovery should be similar to those seen in traditional rehabilitation programs that are widely recognized to lower the risk of institutional care and long-term impairment and raise independence in daily life activities (Kalra & Langhorne, 2007; Pollock et al., 2014). Improvements in function following treatment programs ending have been related to physiological recovery and brain neural plasticity (Kwakkel et al., 2004).

Given the expansion of this field's research and the potential of telerehabilitation to increase accessibility and quality of rehabilitation services while lowering costs, a review was necessary to evaluate this approach. Additionally, it is important since health providers are increasingly providing telerehabilitation services to patients. Consequently, the purpose of this systematic review and meta-analysis was to investigate the effectiveness of telerehabilitation in improving balance and functional mobility in stroke survivors.

## Methods

### Study Design

This study involved systematic review and meta-analysis conducted in accordance with the Preferred Reporting Items for Systematic Reviews and Meta-analyses (PRISMA) guidelines (Page et al., 2021). The review's protocol was registered in the International Prospective Register of Systematic Reviews (PROSPERO) with a registration number (CRD42022306410).

### Search Strategy

Systematic searching for literature was performed from inception to May 2022. The initial search was done via the following databases and registers: PubMed, MEDLINE, Cochrane Central Register of Controlled Trials (CENTRAL), Physiotherapy Evidence Database (PEDro), Wiley Online Library, EBSCO, ScienceDirect, REHABDATA, and an Randomized Controlled Trial (RCT) registration website (http://www.ClinicalTrials.gov). Furthermore, searching was done via other methods including two grey literature databases (Grey Literature Report and Open Grey), website (ResearchGate), and reference lists of all eligible studies as well as citation searching using Google Scholar. Searching in different databases was performed by two independent reviewers (NA and AA).

The search was performed by using a combination of medical subjective headings (MeSH) and/or keywords related to telerehabilitation, telemedicine, telehealth, videoconferencing, and stroke. Search strategy was developed and modified according to each database's specific Boolean criteria. An example of the PubMed search strategy is presented in Table 1.

**Table 1:** Search Strategy (PubMed)

#1	“Telerehabilitation”[Mesh] OR “Telemedicine”[Mesh] OR “Videoconferencing”[Mesh].
#2	“Stroke”[Mesh]
#3	“Randomized Controlled Trial” [Publication Type] OR “Clinical Trial” [Publication Type]
#4	1 AND 2 AND 3

### Eligibility Criteria

**Figure 1:**
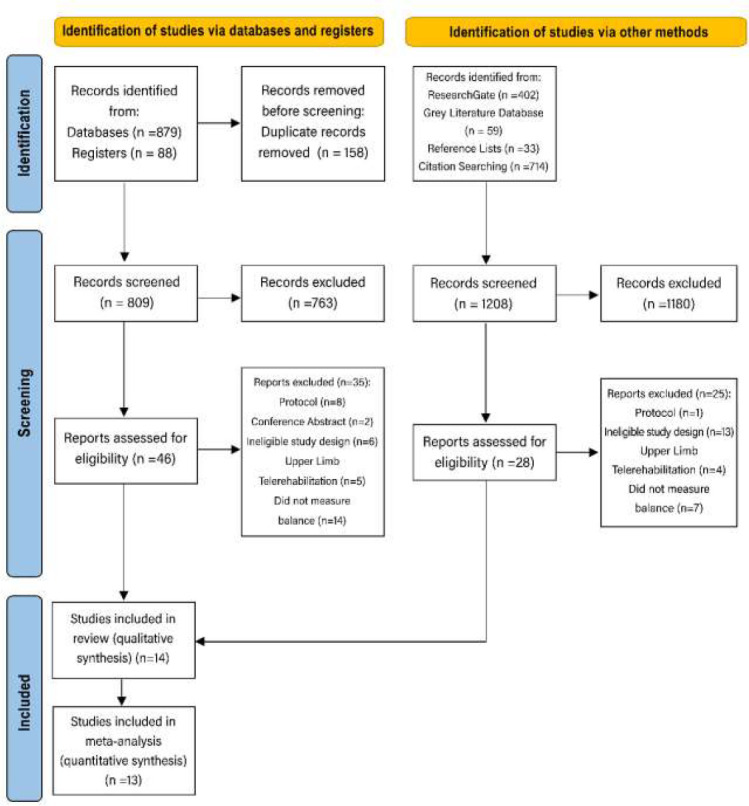
Flow Diagram of Study Selection Process

Studies were eligible for inclusion if all the following PICOS criteria were met: (P) Stroke survivors (aged > 18 years, no restriction based on the type of stroke, sex, or race). An Intervention group (I) received telerehabilitation program (no limitation based on type of technology used) in conjunction with other therapy or alone. A control group (C) did not receive any type of telerehabilitation. The outcomes of interest (O) were balance as a primary outcome, and functional mobility as secondary outcome. The study design (S) was randomized controlled trials, pilot trials, or clinical trials with a control group that were published in English with available full text. Studies that did not meet the previous criteria were excluded. The number of articles and the cause for exclusion are presented in Figure 1.

### Study Selection Process

All records identified through searching were imported into reference management software (EndNote 20). The duplicates were removed, and an initial review of titles and abstracts was done by two independent reviewers. If the abstract did not include sufficient information, full text was screened. After that, for all studies that initially matched the inclusion criteria, a full-text careful review for final decision was performed. Lastly, reference lists and citation searching of all included articles were performed to find further eligible studies. Any discrepancies between the two reviewers (NA and AA) were resolved by a third reviewer (MA) in a consensus meeting.

### Data Extraction, Synthesis and Analysis

In this review, both qualitative and quantitative synthesis were conducted in accordance with PRISMA guidelines (Page et al., 2021), and outcomes of interest were balance (primary outcome) and functional mobility (secondary outcomes).

### Data Extraction

A custom data extraction form was used to extract the data from each included study. Data extraction was independently performed by two reviewers (NA and AA) and reviewed by a third reviewer (AS). The following data were extracted: authors' names, publication year, participants' characteristics (sample size, sex, age), outcome measures used, telerehabilitation and control interventions (type, frequency, duration of sessions and program), follow-up, and main results. These data are represented in Table 2.

In order to conduct meta-analysis, the sample size, mean, and standard deviation of the telerehabilitation and control groups were extracted. If any study results were not reported in form of mean and standard deviation, the study's authors were contacted to obtain those data.

**Table 2:** Characteristics of Included Studies

Study	Sample characteristics	Measured variables	Intervention	Follow-up	Summary of results (p-value)
(Cikajlo et al., 2012)	Sample size= 26 (M=14, F=12) Age: ^*^ TRG= 58.5 ± 12.1 CG= 61 ± 7.4	Berg Balance Scale (BBS) Timed Up & Go Test (TUG) 10 Meter Walk Test (10MWT)	**TRG:** Virtual reality-based balance training with standing frame under therapist supervision who guide the training remotely via videoconference. **CG:** Balance training with standing frame, but without VR in the clinic. Five 20-min sessions/week for almost 3 weeks in TRG and 4 weeks in CG	2 weeks	Both groups improved significantly on BBS, TUG, and 10MWT scores (P < 0.05), and the effect was preserved at follow-up. However, no significant between-group differences were detected in all scales (P > 0.05).
(Krpic et al., 2013)	Sample size=26 (M=15, F=11) Age: ^*^ TRG= 58.5±12.1 CG= 63.0±8.5 AG= 61.0±7.4	Berg Balance Scale (BBS) Timed Up & Go Test (TUG) 10 Meter Walk Test (10MWT)	**TRG:** Virtual reality supported balance training by using balance frame and large screen displayed tasks and instructions, therapist provided verbal instructions on how to correct the posture, hand placement, etc. via videoconferencing. **CG:** Conventional balance training with various objects (e.g., ball) in hospital. **AG:** Balance training with balance frame (holding a ball and leaning in different directions, etc.) in hospital. Five sessions/week for almost 3 weeks in TRG and 4 weeks in CG & AG. Sessions' duration was 15-min in TRG, 45-min in CG, and 20-min in AG.	No	BBS, TUG, and 10MWT scores improved significantly in all groups (P<0.05). Nonetheless, there was no significant difference between the groups (P > 0.05).
(Lin et al., 2014)	Sample size= 24 (M=17, F=7) Age: ^*^ TRG= 78.6±7.96 CG= 75.6±11.77	Berg Balance Scale (BBS) Barthel Index (BI) – Mobility Domains	**TRG:** A WSN telerehabilitation system was used to conduct the balance training program (including static/dynamic sitting balance, touch screen manipulation, etc.) instructed by a remote therapist via videoconferencing. This system also involves vital signs monitor. **CG:** Conventional balance training program (including static/dynamic sitting balance, ball manipulation, etc.) instructed by a therapist via personal face-to-face contact. Three 50-min sessions/week for 4 weeks	No	Both groups showed significant improvement on BBS score (P < 0.001) and non-significant change in BI-Mobility (p= 0.088). In addition, no significant between-group differences were observed in all outcomes (P > 0.05).
(Lloréns et al., 2015)	Sample size= 30 (M=17, F=13) Age: ^*^ TRG= 55.47±9.63 CG= 55.6±7.29	Berg Balance Scale (BBS) Performance-Oriented Mobility Assessment - Balance (POMA-B) Brunel Balance Assessment (BBA)	**TRG:** Virtual reality-based telerehabilitation training program. The virtual environment involved a central circle and various items placed around it. Participants were asked to step on these items with the nearest foot while maintaining the other foot within the circle. There are nine defined levels of difficulty based on configuring the location of appearance, etc. **CG:** In-clinic virtual reality-based training program. Same procedures as TRG. # Both groups received conventional physical therapy in the clinic Three 45-min sessions for 8 weeks and a total of 20 sessions. At same time, both groups received conventional physiotherapy sessions twice/week.	4 weeks	A significant effect was detected in both groups in all outcomes at post-intervention (P<0.01), these improvements were preserved as non-significant differences were detected from post-intervention to the follow-up. However, no significant between-group differences were detected on any scale (P > 0.05).
(van den Berg et al., 2016)	Sample size= 63 (M=40, F=23) Age: ^*^ TRG= 64.5 ± 18.5 CG= 70.1 ± 12.4	Stroke Impact Scale (SIS) – Mobility Domain Berg Balance Scale (BBS) Timed Up & Go Test (TUG) 10 Meter Walk Test (10MWT) Rivermead Mobility Index (RMI)	**TRG:** Usual rehabilitation care and Caregiver-mediated training program with a support of a customized exercise application (includes gait, standing, turning, etc.) and videoconferencing with the treating physiotherapist. Also, to increase patients' physical activity, they wore a Fitbit Zip activity tracker. **CG:** Usual care received interdisciplinary rehabilitation (addressing mobility impairment, upper limb activity, sensorimotor impairment, ADL, etc.) Five 30-min sessions/week for 8 weeks	4 weeks	At post-intervention, no significant between-group differences were observed in SIS – Mobility, BBS, 10MWT, TUG, and RMI (P > 0.05). At follow-up, no significant between-group differences were observed in all outcome measures except TUG as there was a significant effect of CG (P=0.03).
(Chen et al., 2017)	Sample size= 54 (M=33, F=21) Age: ^*^ TRG= 66.52 ± 12.08 CG= 66.15 ± 12.33	Berg Balance Scale (BBS)	**TRG:** Home-based tele-supervising rehabilitation including physical exercises (balance exercises, walking exercises, etc.) and electromyography-triggered neuromuscular stimulation (ETNS). Therapists supervised the participants by live videoconferencing. **CG:** Physical exercises and ETNS program carried out face-to-face in the outpatient rehabilitation department. 1-hour physical exercises and 20-min ETNS, twice in a working day for 12 weeks and a total of 60 sessions	12 weeks	Both groups demonstrated a significant effect within groups in improving BBS over time (P<0.001), but no significant between-group differences was observed (p > 0.05).
(Vloothuis et al., 2019)	Sample size= 66 (M=41, F=25) Age: ^*^ TRG= 60.53 ± 14.82 CG= 59.26 ± 15.01	Stroke Impact Scale (SIS) – Mobility Domain Berg Balance Scale (BBS) Timed Up & Go Test (TUG) 10 Meter Walk Test (10MWT) 6-Minute Walking Test (6MWT) Rivermead Mobility Index (RMI) Nottingham Extended ADL Scale – Mobility Domain	**TRG:** Usual care and CARE4STROKE program (caregiver-mediated exercises with e-health application include 37 standardized exercises aimed at improving mobility and telerehabilitation services like telephone, videoconferencing, etc.) **CG:** Usual care included exercises to improve standing balance, physical condition, walking competence, etc. Five 30-min sessions/week for 8 weeks	4 weeks	No significant between-group differences were found in all outcome measures at post-intervention and follow-up (p > 0.05).
(Burgos et al., 2020)	Sample size= 10 (M=6, F=4) Age: ^*^ TRG= 57±6.63 CG= 67.75±8.3	Berg Balance Scale (BBS) Mini Balance Evaluation Systems Test (Mini-BEST)	**TRG:** Standard rehabilitation treatment at the hospital and telerehabilitation balance training by using smartphone-based exergames controlled by body motions. Physiotherapist monitored patients ' scores by connecting to the web platform to keep standard interaction and increase exercise dose when needed. **CG:** Standard rehabilitation treatment at the hospital. Both groups received standard rehabilitation three 40-min sessions/week for 4 weeks. At same time, TRG received additional telerehabilitation nine 30-min sessions/week.	No	The results show a significant improvement in the BBS and Mini-BEST for the TRG. However, only the improvement of BBS was statistically higher for the TRG compared to the CG (P < 0.05).
(Wu et al., 2020)	Sample size= 61 (M=36, F=25) Age: ^*^ TRG= 56.73±11.85 CG= 59.10±8.60	Berg Balance Scale (BBS) Timed Up & Go Test (TUG) 6-Minute Walking Test (6MWT)	**TRG:** During the hospitalization, patients received early rehabilitation and nursing routine included (normal limb position, bed position, transfer, etc.). After discharge, patients received home remote rehabilitation based on a multi-disciplinary care model that was applied via videoconferencing system. The intervention included sitting-up training, balance training, etc. **CG:** Received the same intervention as the TRG during hospitalization. After discharge, rehabilitation guidance was conducted by telephone once a week, and rehabilitation clinic visited to get instructions as needed. Twelve weeks	No	Both groups were significantly improved in BBS, TUG, and 6MWT (P < 0.05), but the TRG showed statistically significant greater improvement.
(Chen et al., 2021)	Sample size= 30 (M=18, F=12) Age: ^**^ TRG= 61 (53–68) CG= 60 (52–68)	Berg Balance Scale (BBS) Timed Up & Go Test (TUG) Functional Ambulation Category (FAC)	**TRG:** Telerehabilitation using a Kinect camera-based interactive system. The program included target-oriented stepping tasks, multidirectional reaching tasks, and Tai Chi exercises. **CG:** One-on-one conventional training sessions included sitting to standing movements, balance exercises, standing, etc. Three 40-min sessions/week for 4 weeks	No	Group comparison showed no significant differences in BBS, TUG, and FAC scores (p > 0.05). However, BBS scores improved significantly in both groups while TUG improved significantly in the TRG only.
(Junata et al., 2021)	Sample size= 30 (M=24, F=6) Age: ^*^ TRG= 60.6 ± 5.5 CG= 60.1 ± 5.8	Berg Balance Scale (BBS) Timed Up & Go Test (TUG) Lean-and-Release Assessment	**TRG:** Kinect-based rapid movement training platform. Training included arms and legs movement in 22 different and randomized directions as quickly and as far as possible. **CG:** Conventional balance training (sit-to-stand, lateral stepping, etc.) Three 60-min sessions/week for seven weeks, and a total of 20 sessions.	No	Both groups improved significantly on BBS score, but TUG score improved significantly only in TRG (P < 0.05). Groups comparison showed no significant differences in all outcomes (p > 0.05), except for step displacement and step length (P < 0.05).
(Saywell et al., 2021)	Sample size= 95 (M=49, F=46) Age: ^*^ TRG= 74.1±11.7 CG= 72.9±11.7	Step Test Stroke Impact Scale (SIS) – Mobility Domain	**TRG:** Augmented community telerehabilitation intervention (ACTIV) focused on 2 functional categories: “staying upright” and “using your arm”. Exercises and activities were selected based on patients ' goals. Each participant received 4 face-to-face visits, 5 structured phone calls, and personalized text messages. **CG:** Usual Care (no further formal rehabilitation) 6 months	6 months	No significant between groups differences were observed in Step Test & SIS – Mobility Domain at post-intervention and follow-up (p > 0.05).
(Salgueiro et al., 2022a)	Sample size=30 (M=20, F=10) Age: ^*^ TRG= 57.27 ±14.35 CG= 64.53 ± 9.40	Postural Assessment Scale for Stroke Patients (PASS) Berg Balance Scale (BBS)	**TRG:** Conventional physiotherapy and home-based core-stability exercises (pelvic tilt, bridging, trunk rotation, etc.) guided by a telerehabilitation app. **CG:** Conventional physiotherapy consisted of stretching, mobilization, aerobic training, etc. conducted by a therapist via personal face-to-face contact. Both groups received conventional physiotherapy two 60-min sessions/week for 12 weeks. At same time, TRG received additional telerehabilitation five sessions/week.	No	PASS improved significantly in TRG only (P<0.05), while BBS improved significantly in both TRG and CG (P<0.05). However, there were no significant differences between groups (p > 0.05).
(Salgueiro et al., 2022b)	Sample size= 49 (M=31, F=18) Age: ^*^ TRG= 71.58±10.72 CG= 70.68±14.08	Postural Assessment Scale for Stroke Patients (PASS) Berg Balance Scale (BBS)	**TRG:** Usual care and telerehabilitation via Farmalarm app which include core-stability exercises and videoconference with an experienced neurological physiotherapist. **CG:** Usual care consisted of therapeutic techniques such as muscle stretching, passive and functional mobilization of the affected body segments, balance exercises and gait training. Average of 2.5 sessions/week for three months	No	No significant between-group differences were found in PASS and BBS at post-intervention (p > 0.05).

*Note*. ^*^ Values are expressed as mean ± standard deviation (SD); ** Values are expressed as median (interquartile range (IQR)); **TRG:** Telerehabilitation group, **CG:** control/comparison group, **AG:** Additional group, **M:** Male, **F:** Female, **BBS:** Berg Balance Scale, **TUG:** Timed Up & Go Test, **10MWT:** 10 Meter Walk Test, **BI:** Barthel Index, **POMA-B:** Performance-Oriented Mobility Assessment - Balance, **BBA:** Brunel Balance Assessment, **SIS:** Stroke Impact Scale, **RMI:** Rivermead Mobility Index, **6MWT:** 6-Minute Walking Test, **Mini-BEST:** Mini Balance Evaluation Systems Test, **FAC:** Functional Ambulation Category, **PASS:** Postural Assessment Scale for Stroke Patients, **ADL:** Activities of Daily Living.

### Meta-analysis

Review Manager (version 5.4) software was used to conduct the meta-analysis and generate forest plots. Overall effect size with 95% confidence interval was calculated using random-effects model. Standard mean difference (SMD) was used as effect measure and its clinical significance was interpreted according to Cohen's effect size classifications: small (0.2), medium (0.5), and large (0.8) (Cohen, 2013). In addition,*I*^2^ statistic was used to assess variability (heterogeneity) across trials, and results were rated according to the following classifications: low (*I*^2^ ≤ 25%), medium (*I*^2^ 26 – 50%), and high (*I*^2^ ≥ 75%) (Huedo-Medina et al., 2006).

Moreover, subgroup analyses were also conducted to assess the effectiveness of telerehabilitation alone or combined with conventional rehabilitation versus various control interventions.

### Sensitivity Analysis

Two sensitivity analyses were conducted to evaluate the robustness of the results: (1) omitting outlier studies that had a confidence interval does not overlap with the confidence interval of the pooled effect (Viechtbauer & Cheung, 2010), (2) excluding studies with a poor methodological quality.

### Publication Bias

SPSS (version 28) was used to assess the publication bias when the meta-analysis included 10 or more studies (Sterne et al., 2011). Evaluation was done by visual inspection of funnel plot and Egger's test which is used to test funnel plot asymmetry (Egger et al., 1997). In presence of publication bias, the trim and fill method was used to adjust the overall effect size based on missing studies (Duval & Tweedie, 2000).

### Risk of Bias Assessment

PEDro scale was used to assess the included studies' methodological quality. It was developed by the Physiotherapy Evidence Database (PEDro) (Maher et al., 2003), and it is a reliable scale that is commonly used in systematic reviews (da Costa et al., 2013). There are 11 items on this scale with a total score of 10 as criteria one is not included in the overall score (Maher et al., 2003). The following criteria were used to rate the included studies: excellent quality (9–10), good quality (6–8), fair quality (4–5), or poor quality (≤3) (Foley et al., 2003). Two independent reviewers (NA and AS) assessed the methodological quality, and any disagreements between the two reviewers were resolved by a third reviewer (AE).

### Quality of Evidence Assessment

The assessment of quality of evidence was performed by two reviewers (MA and AF). The Grading of Recommendations Assessment, Development, and Evaluation (GRADE) system was used to assess certainty of evidence and make a summary of findings table. There are four levels for rating evidence: ‘High quality’, ‘Moderate quality’, ‘Low quality’ or ‘Very low quality’. Each of these levels has a description that focuses on the certainty of the results, how much the effect estimate is closely related to the true effect and the influence of evidence degree for recommending future studies. GRADE evaluation was determined through consideration of five domains (risk for bias, inconsistency, indirectness, imprecision, and publication bias) (Atkins et al., 2004; Balshem et al., 2011; Guyatt et al., 2011).

## RESULTS

### Study Selection

In the initial search of databases and registers, 967 records were identified. After deletion of 158 duplicated records, 809 records were screened by title and abstract, and 763 ineligible records were excluded. Of the 46 studies reviewed in full text, only 11 met the inclusion criteria (Burgos et al., 2020; Chen et al., 2017; Chen et al., 2021; Cikajlo et al., 2012; Krpic et al., 2013; Lin et al., 2014; Lloréns et al., 2015; Salgueiro et al., 2022a; Saywell et al., 2021; Vloothuis et al., 2019; Wu et al., 2020). Furthermore, 1208 records were identified through searching via other methods. After exclusion of 1180 records, 28 records were assessed for eligibility and only three studies met the inclusion criteria (Junata et al., 2021; Salgueiro et al., 2022b; van den Berg et al., 2016). Consequently, fourteen articles were included in the review, and thirteen of them were included in the meta-analysis (Burgos et al., 2020; Chen et al., 2017; Cikajlo et al., 2012; Junata et al., 2021; Krpic et al., 2013; Lin et al., 2014; Lloréns et al., 2015; Salgueiro et al., 2022a; Saywell et al., 2021; van den Berg et al., 2016; Vloothuis et al., 2019; Wu et al., 2020). The PRISMA flow diagram was used to illustrate the searching steps, selection procedures, and exclusion reasons (Figure 1).

### Study Characteristics

Fourteen studies with a total of 594 participants were included in this review, 278 participants were in telerehabilitation groups and 316 in control groups. Sample size ranged from 10 to 95 and included both genders. All included studies were published during the period from 2012 to 2022.

Balance was evaluated in all included studies by using different outcome measures, such as Berg Balance Scale (BBS), Performance-Oriented Mobility Assessment - Balance (POMA-B), Brunel Balance Assessment (BBA), Mini Balance Evaluation Systems Test (Mini-BEST), Lean-and-Release Assessment, and Step Test. While mobility and gait were assessed by using 10 Meter Walk Test (10MWT), Rivermead Mobility Index (RMI), 6-Minute Walking Test (6MWT), Functional Ambulation Category (FAC), Mobility Domains of Barthel Index (BI), Stroke Impact Scale (SIS), and Nottingham Extended ADL Scale (NEADL). Moreover, Timed Up & Go Test (TUG) and Postural Assessment Scale for Stroke Patients (PASS) were used to measure both balance and mobility. Monitoring and follow-up periods ranged from two weeks to 12 months in six studies (Chen et al., 2017; Cikajlo et al., 2012; Lloréns et al., 2015; Saywell et al., 2021; van den Berg et al., 2016; Vloothuis et al., 2019), while other studies did not follow up the patients.

The intervention groups received telerehabilitation in conjunction with conventional rehabilitation in six studies (Burgos et al., 2020; Lloréns et al., 2015; Salgueiro et al., 2022a, 2022b; van den Berg et al., 2016; Vloothuis et al., 2019), while the other eight studies focused only on telerehabilitation (Chen et al., 2017; Chen et al., 2021; Cikajlo et al., 2012; Junata et al., 2021; Krpic et al., 2013; Lin et al., 2014; Saywell et al., 2021; Wu et al., 2020). Different therapies were given via a variety of telerehabilitation systems, but video and audio equipment for videoconferencing capabilities were the most common.

On the other hand, control groups' interventions were conventional rehabilitation (Burgos et al., 2020; Chen et al., 2017; Chen et al., 2021; Salgueiro et al., 2022a, 2022b; Saywell et al., 2021; van den Berg et al., 2016; Vloothuis et al., 2019; Wu et al., 2020), balance training (Cikajlo et al., 2012; Junata et al., 2021; Krpic et al., 2013; Lin et al., 2014), and virtual reality based training plus conventional rehabilitation (Lloréns et al., 2015). Duration of the training program varied and lasted from 3 weeks to 6 months. Characteristics of the included studies were summarized in Table 2. Data synthesis

A descriptive synthesis was performed for all included studies and each study's findings are presented in Table 2. Different balance outcome measures were assessed in the included studies, however, the most measured balance outcome was BBS as it was evaluated in thirteen studies (Burgos et al., 2020; Chen et al., 2017; Chen et al., 2021; Cikajlo et al., 2012; Junata et al., 2021; Krpic et al., 2013; Lin et al., 2014; Lloréns et al., 2015; Salgueiro et al., 2022a, 2022b; van den Berg et al., 2016; Vloothuis et al., 2019; Wu et al., 2020), two studies found that there was a significant improvement in telerehabilitation group compared to control (Burgos et al., 2020; Wu et al., 2020), while the other eleven studies found a non-significant difference between groups. Moreover, three studies evaluated other balance outcome measures including POMA-B, BBA, Mini-BEST, and step test (Burgos et al., 2020; Lloréns et al., 2015; Saywell et al., 2021), and reported non-significant between groups difference.

Assessment of functional mobility was done in six studies (Chen et al., 2021; Lin et al., 2014; Salgueiro et al., 2022a; Saywell et al., 2021; van den Berg et al., 2016; Vloothuis et al., 2019) by using various outcomes, such as RMI, FAC, Mobility Domains of BI, SIS, NEADL, and PASS, however, all six studies reported non-significant between groups difference in terms of improving functional mobility. Furthermore, evaluation of balance and mobility by using TUG was done in six studies (Chen et al., 2021; Cikajlo et al., 2012; Junata et al., 2021; Krpic et al., 2013; van den Berg et al., 2016; Vloothuis et al., 2019; Wu et al., 2020), all studies reported a non-significant between groups difference, except (Wu et al., 2020), which found significantly greater improvement in telerehabilitation group compared to control.

### Risk of Bias in Studies

According to the PEDro scale, included studies' methodological quality values varied from 3 to 8 out of 10. Ten studies were rated as good quality (Burgos et al., 2020; Chen et al., 2017; Junata et al., 2021; Lin et al., 2014; Lloréns et al., 2015; Salgueiro et al., 2022a; Saywell et al., 2021; van den Berg et al., 2016; Vloothuis et al., 2019; Wu et al., 2020), three studies were rated as fair quality (Chen et al., 2021; Krpic et al., 2013; Salgueiro et al., 2022b), and one study was rated as poor quality (Cikajlo et al., 2012). All included studies did not perform subjects' and therapists' blinding. In addition to these criteria, the least met criteria were allocation concealment (six studies), assessors' blinding (nine studies), and intention-to-treat analysis (nine studies). Thus, the highest risk of bias for these studies was selection, performance, detection, and attrition bias. Details of the methodological quality appraising of the included studies are shown in Table 3.

### Meta-analysis

#### Effects of Telerehabilitation on Balance

**Figure 2:**
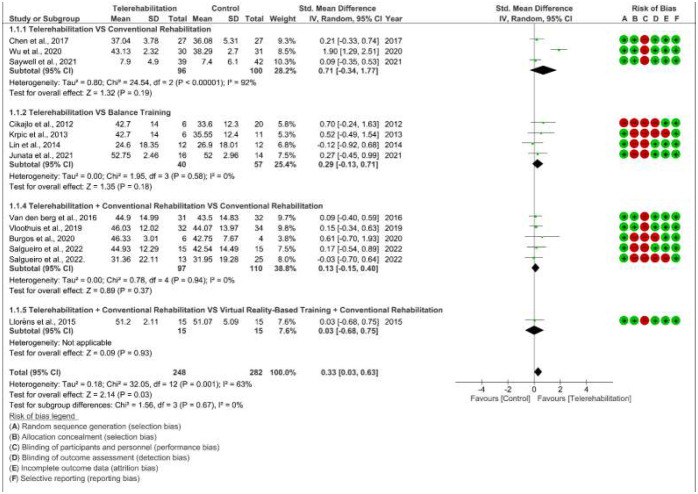
Forest Plot for the Effect of Telerehabilitation Alone or Combined with Conventional Rehabilitation Versus Various Control Interventions on Balance

Thirteen studies with 530 participants were eligible to be included in this analysis (Burgos et al., 2020; Chen et al., 2017; Cikajlo et al., 2012; Junata et al., 2021; Krpic et al., 2013; Lin et al., 2014; Lloréns et al., 2015; Salgueiro et al., 2022a, 2022b; Saywell et al., 2021; van den Berg et al., 2016; Vloothuis et al., 2019; Wu et al., 2020), and one study (Chen et al., 2021) was excluded because results were expressed in form of median and interquartile range. The overall effect was favorable for telerehabilitation in terms of improving balance in stroke survivors with a significant difference and small effect size (SMD 0.33 [95% CI 0.03 to 0.63]; P =0.03). In addition, variability across studies evaluation showed presence of medium heterogeneity (*I*^2^ = 63%) (Figure 2).

Overall, subgroup analysis showed non-significant favor toward telerehabilitation with presence of small to medium effect sizes. Furthermore, the subgroup differences test revealed a non-significant subgroup effect (P = 0.67) (Figure 2).

Sensitivity analysis was performed by excluding Wu et al., (2020) which had a larger effect size than other studies in the meta-analysis and was considered an outlier study. Even after removing this outlier study, telerehabilitation was still associated with a small effect size in improving balance, however the result was no longer significant (SMD 0.15 [95% CI −0.03 to 0.34]; P =0.1). Moreover, excluding Wu et al., (2020) had a significant effect on reducing the heterogeneity across studies (*I*^2^ = 0%) (Appendix A).

Another sensitivity analysis was conducted by omitting (Cikajlo et al., 2012) which had poor methodological quality. Upon removal of this study, the effect size barely changed, but the result was no longer significant (SMD 0.31 [95% CI −0.01 to 0.63]; P =0.06). However, heterogeneity across studies did not reduce after excluding (Cikajlo et al., 2012) (*I*^2^ = 65%) (Appendix B).

#### Effects of Telerehabilitation on Functional Mobility

**Figure 3:**
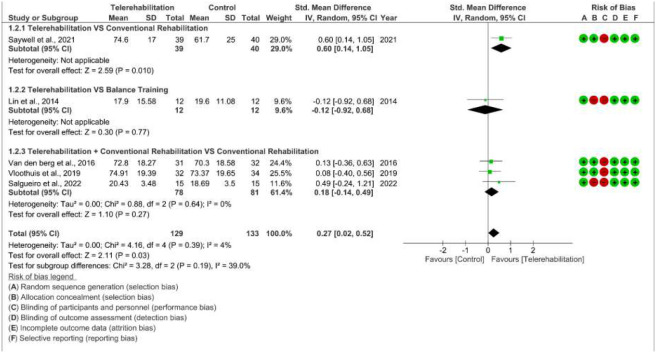
Forest Plot for the Effect of Telerehabilitation Alone or Combined with Conventional Rehabilitation versus Various Control Interventions on Functional Mobility

Five studies with 262 participants were eligible to be included in this analysis (Lin et al., 2014; Salgueiro et al., 2022a; Saywell et al., 2021; van den Berg et al., 2016; Vloothuis et al., 2019). The overall effect was favorable for telerehabilitation in terms of improving functional mobility in stroke survivors with a significant difference and small effect size (SMD 0.27 [95% CI 0.02 to 0.52]; P =0.03). Moreover, variability across studies evaluation showed presence of low heterogeneity (*I*^2^ = 4%) (Figure 3).

Subgroup analysis showed a significant medium effect size of telerehabilitation when compared to conventional rehabilitation (SMD 0.6; P=0.01), and a non-significant small effect size of telerehabilitation plus conventional rehabilitation when compared to conventional rehabilitation (SMD 0.18; P=0.27), while balance training showed a non-significant small effect when compared to telerehabilitation (SMD −0.12; P=0.77). However, subgroup differences test showed non-significant subgroup effect (P = 0.19) (Figure 3).

Since no studies of poor methodological quality were included in this meta-analysis and there were no outlier studies, a sensitivity analysis was not conducted.

**Table 3:** Methodological quality assessment of included studies (PEDro scale)

Study	Items											Total score for each study (10)	Level of Quality
	Eligibility criteria *	Random allocation	Concealed allocation	Baseline comparability	Blind subjects	Blind therapists	Blind assessors	Adequate follow-up	Intention-to-treat analysis	Between-group comparisons	Point estimates and variability		
(Cikajlo et al., 2012)	Y	N	N	N	N	N	N	Y	N	Y	Y	3	Poor
(Krpic et al., 2013)	Y	Y	N	Y	N	N	N	N	N	Y	Y	4	Fair
(Lin et al., 2014)	Y	Y	N	Y	N	N	Y	Y	Y	Y	Y	7	Good
(Lloréns et al., 2015)	Y	Y	Y	Y	N	N	Y	Y	Y	Y	Y	8	Good
(van den Berg et al.,	Y	Y	Y	Y	N	N	Y	Y	Y	Y	Y	8	Good
(Chen et al., 2017)	N	Y	Y	Y	N	N	Y	Y	Y	Y	Y	8	Good
(Vloothuis et al., 2019)	Y	Y	Y	Y	N	N	Y	Y	Y	Y	Y	8	Good
(Burgos et al., 2020)	Y	Y	N	Y	N	N	N	Y	Y	Y	Y	6	Good
(Wu et al., 2020)	N	Y	Y	Y	N	N	Y	Y	N	Y	N	6	Good
(Chen et al., 2021)	Y	Y	N	Y	N	N	N	Y	N	Y	Y	5	Fair
(Junata et al., 2021)	Y	Y	N	Y	N	N	Y	Y	Y	Y	Y	7	Good
(Saywell et al., 2021)	Y	Y	Y	Y	N	N	Y	N	Y	Y	Y	7	Good
(Salgueiro et al., 2022a)	Y	Y	N	Y	N	N	Y	Y	Y	Y	Y	7	Good
(Salgueiro et al., 2022b)	Y	Y	N	Y	N	N	N	N	N	Y	Y	4	Fair
Total score for each item	12	13	6	13	0	0	9	11	9	14	13		

#### Publication Bias

**Figure 4:**
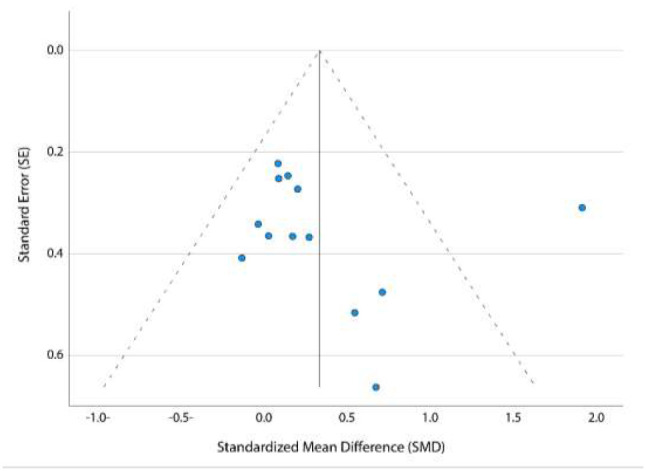
Funnel Plot for the Meta-analysis of the Effect of Telerehabilitation on Balance

Visual inspection of the funnel plot showed slight asymmetry, an indication that publication bias may be present (Figure 4). However, a non-significant Egger's test indicated absence of publication bias (p=0.9). Additionally, the overall effect size was not modified by the ‘trim and fill’ method, demonstrating that there was no need for adjustment based on missing studies.

#### Quality of Evidence

According to the GRADE system, the quality of evidence was rated from ‘Low’ quality to ‘Very Low’ quality due to the presence of a serious or very serious risk of bias, inconsistency, or imprecision. Table 4 includes further details on the GRADE evaluation.

**Table 4:** Quality of evidence (GRADE)

*Outcome measured*	*Intervention*	*N. of part. (studies)*	*Study limitation*	*Inconsistency*	*Indirectness*	*Imprecision*	*Publication bias*	*Overall quality of evidence*	*Effect Estimate SMD [95% CI]*	*Effect size*	*Direction*
**Balance**	**Overall**	530 (13)	Very Serious^a^	Not Serious	Not Serious	Not Serious	Not Serious	⊕⊕◯◯ Low	0.33 [0.03, 0.63]	Small	Favor to TR
	**TR VS CR**	196 (3)	Serious^b^	Serious^c^	Not Serious	Serious^d^	Not Serious	**⊕◯◯◯** Very Low	0.71 [-0.34, 1.77]	Medium	Favor to TR
	**TR VS BT**	97 (4)	Very Serious^a^	Not Serious	Not Serious	Serious^d^	Not Serious	**⊕◯◯◯** Very Low	0.29 [-0.13, 0.71]	Small	Favor to TR
	**TR + CR VS CR**	207 (5)	Very Serious^a^	Not Serious	Not Serious	Serious^d^	Not Serious	**⊕◯◯◯** Very Low	0.13 [-0.15, 0.40]	Small	Favor to TR
	**TR + CR VS VR + CR**	30 (1)	Serious^b^	Not Serious	Not Serious	Serious^d^	Not Serious	⊕⊕◯◯ Low	0.03 [-0.68, 0.75]	Small	Favor to TR
***Functional Mobility***	**Overall**	262 (5)	Serious^b^	Not Serious	Not Serious	Serious^d^	Not Serious	⊕⊕◯◯ Low	0.27 [0.02, 0.52]	Small	Favor to TR
	**TR VS CR**	79 (1)	Serious^b^	Not Serious	Not Serious	Serious^d^	Not Serious	⊕⊕◯◯ Low	0.60 [0.14, 1.05]	Medium	Favor to TR
	**TR VS BT**	24 (1)	Very Serious^a^	Not Serious	Not Serious	Serious^d^	Not Serious	**⊕◯◯◯** Very Low	-0.12 [-0.92, 0.68]	Small	Favor to BT
	**TR + CR VS CR**	159 (3)	Serious^b^	Not Serious	Not Serious	Serious^d^	Not Serious	⊕⊕◯◯ Low	0.18 [-0.14, 0.49]	Small	Favor to TR

*Note.* **GRADE:** Grading of Recommendations Assessment, Development, and Evaluation; **SMD:** Standardized Mean Difference; **CI:** Confidence Interval; **TR:** Telerehabilitation; **CR:** Conventional Rehabilitation; **BT:** Balance Training; **VR:** Virtual Reality-Based Training.

**Reasons to downgrade the current evidence:**

a Crucial limitation for more than one criterion;

b Crucial limitation for one criterion;

c High heterogeneity *I*^2^ > 75%;

d Small sample size.

⊕⊕◯◯: Low quality (limited confidence in the estimated effect); ⊕◯◯◯: Very Low quality (very limited confidence in the estimated effect).

#### Discussion

The purpose of this systematic review and meta-analysis was to investigate the effectiveness of telerehabilitation in improving balance and functional mobility in stroke survivors. The results showed that telerehabilitation was associated with a significant but small improvement in balance (SMD 0.33; low-quality evidence) and functional mobility (SMD 0.27; low-quality evidence) immediately post-intervention in stroke survivors. The methodological quality of the included studies was rated as good quality in ten studies, fair quality in three studies, and poor quality in one study.

Improving balance is one of the most important rehabilitation aspects that should be targeted during rehabilitation programs for stroke survivors to improve their safe mobility and overall function (Wu et al., 2020). This may require a long-term course of rehabilitation that can be delivered via telerehabilitation, which is an effective way to allow easier access to physiotherapy for patients with serious disabilities (Peretti et al., 2017). In addition, previous studies reported that telerehabilitation could decrease the costs of treating and rehabilitating patients (Nizeyimana et al., 2022; Perry et al., 2011). Unfortunately, a number of obstacles prevent telerehabilitation from being widely used, such as administrative license, medicolegal ambiguity, financial stability, and absence of technology infrastructure particularly in low-income countries (Akbik et al., 2017; Sarfo et al., 2017). To get through these obstacles, telerehabilitation systems need to be developed and studied more in the future.

The findings of this review suggest that telerehabilitation could help stroke survivors improve their balance and functional mobility. However, there is a significant variation amongst the included studies in terms of the interventions utilized, the comparison interventions, and the outcomes measured. In addition, different information and communication technologies were used; for example, some research relied on telephone calls, while others included videoconferencing, mobile apps, and virtual reality systems. Furthermore, the length of rehabilitation programs and the frequency of follow-up or interactions with medical staff varied from one study to the next. There is currently insufficient evidence in the literature to determine which model or telerehabilitation tool is best for these individuals, and future comparative studies are recommended.

Subgroup analyses were carried out to assess the impact of various interventions used in the included studies. However, neither of the analyses proved that the effects of the various subgroups varied. The possible explanation was the presence of a non-significant small effect of telerehabilitation alone or combined with conventional rehabilitation on balance and functional mobility when compared with various control interventions in most of the included studies.

Funnel plot asymmetry and the presence of medium statistical heterogeneity among trials in the balance meta-analysis could be due to the existence of outlier study and methodological quality issues (Egger et al., 1997). According to the sensitivity analyses, the overall significant effect of telerehabilitation on balance was affected by removing the outlier and poor methodological quality studies. However, none of these analyses changed the direction of the effect, with the greatest decrease in heterogeneity and effect size when the outlier study was excluded.

Due to the lack of blinding in the subjects, assessors, and therapists, as well as no allocation concealment or intention to treat analysis, there were some methodological issues, including selection, performance, detection, and attrition bias. Moreover, the GRADE system was used to classify the level of evidence, and it revealed low to very low-quality evidence, which limited the confidence in the effect estimate. Additionally, the limited number of trials, the presence of small sample sizes in many included studies, discrepancies in rehabilitation protocols, and the frequency and duration of the interventions across trials, made it is difficult to draw clinical recommendations based on the current evidence.

The current review included fourteen studies investigating the efficacy of telerehabilitation on balance and functional mobility. All of the studies were published within the last 10 years, indicating that this method of rehabilitation is still relatively new. Some ongoing studies have been found, suggesting that more research will be forthcoming. Researchers in this area should perform large RCTs with high methodological quality that could support the available evidence and provide more information, as well as report the data in accordance with a clear standardized guidelines such as CONSORT guidelines (Schulz et al., 2010). This review describes different telerehabilitation application methods and outlines both advantages and disadvantages of this approach, which could be a starting point for improving telerehabilitation's techniques and equipment. It should be noted that these trials should not necessarily show that telerehabilitation produces superior outcomes, but rather proof of comparable outcomes, so it will provide evidence to support the use of this new and alternative method of delivering rehabilitative services that is more affordable and accessible.

This review has some limitations that would limit the generalizability of the findings. Searching included only papers that were published in English. Relevant studies in other languages were neglected, which may have impacted the results and conclusion. Moreover, no analysis or conclusions regarding long-term effects could be made, as several of the reviewed papers did not provide follow-up data. Furthermore, standardized mean difference, which is less clinically significant than a mean difference, was used to compute the pooled effects. Lastly, the accuracy of the reported finding may be impacted due to presence of the outlier study.

#### Conclusion

According to the available evidence, telerehabilitation may improve balance and functional mobility in stroke survivors. However, it is evident that more high-quality research is required due to the existence of low to very low-quality evidence with limited confidence in the effect estimate.

## Author Information

**Nahla Almatrafi:** pt.nahla.ahmad@gmail.com, Tel: +966545659790.

**Abdulqader Almutairi:** s44287239@st.uqu.edu.sa, Tel: +966564427912.

**Amir El Fiky:** aafiky@uqu.edu.sa, phone: +966540728016.

**Ahmed Elsodany:** ahmed_sodany@hotmail.com Tel: 00966530885696.
